# Fetal Alcohol-Related Postnatal Growth Restriction Is Independent of Infant Feeding Practices and Postnatal Alcohol Exposure in a Prospective South African Birth Cohort

**DOI:** 10.3390/nu15092018

**Published:** 2023-04-22

**Authors:** Alexia C. Edwards, Sandra W. Jacobson, Marjanne Senekal, Neil C. Dodge, Christopher D. Molteno, Ernesta M. Meintjes, Joseph L. Jacobson, R. Colin Carter

**Affiliations:** 1 Departments of Emergency Medicine and Pediatrics, Institute of Human Nutrition, Columbia University Vagelos College of Physicians and Surgeons, New York, NY 10032, USA; alexia.edwards@downstate.edu; 2 Adult & Pediatric Intensive Care Units, State University of New York Downstate Medical Center, Brooklyn, NY 11203, USA; 3 Department of Psychiatry and Behavioral Neurosciences, Wayne State University School of Medicine, Detroit, MI 48201, USA; sandra.jacobson@wayne.edu (S.W.J.); neil.dodge@wayne.edu (N.C.D.); joseph.jacobson@wayne.edu (J.L.J.); 4 Department of Human Biology, University of Cape Town Faculty of Health Sciences, Cape Town 7925, South Africa; marjanne.senekal@uct.ac.za (M.S.); ernesta.meintjes@uct.ac.za (E.M.M.); 5 Department of Psychiatry and Mental Health, University of Cape Town Faculty of Health Sciences, Cape Town 7925, South Africa; moltenochris@gmail.com

**Keywords:** breastfeeding, exclusive breastfeeding, fetal alcohol syndrome (FAS), fetal alcohol spectrum disorders (FASD), growth restriction, infant feeding, postnatal alcohol exposure

## Abstract

Prenatal alcohol exposure (PAE) causes growth restriction that worsens in the first year of life. However, the roles of postnatal nutrition in fetal alcohol growth restriction and the impact of postnatal alcohol exposure via breastmilk on growth remain unknown. We aimed to compare infant feeding practices during the first 6.5 months of life between heavy drinkers and abstainers/light drinkers, to examine whether these practices play confounding roles in fetal alcohol growth restriction, and to determine the impact of postnatal alcohol exposure via breastmilk on growth. Eighty-seven heavy-drinking pregnant women and 71 abstainers/light drinkers (controls) were recruited prenatally from antenatal clinics in Cape Town, South Africa. Demographic background and alcohol, cigarette, marijuana, and methamphetamine use during pregnancy were assessed pre- and postnatally. Infant feeding practices were assessed at 6.5 months postpartum using the USDA Infant Feeding Questionnaire. Infant weight, length, and head circumference were measured at 2 weeks, 6.5 and 12 months, and 5 years. Neither prenatal nor postnatal alcohol consumption was related to the duration of breastfeeding, exclusive breastfeeding, exclusive formula, or mixed feeding. Complementary feeding practices were remarkably similar between exposure groups. PAE was related to all postnatal anthropometry measures at all age points, independent of infant feeding practices. Postnatal alcohol exposure via breastmilk was unrelated to any anthropometry outcome after control for PAE. In conclusion, fetal alcohol-related postnatal growth restriction was not attributable to differences in postnatal infant feeding practices or postnatal alcohol exposure and is thus likely a direct teratogenic effect of PAE.

## 1. Introduction

Despite intensive public health campaigns, maternal alcohol consumption during pregnancy continues to be prevalent worldwide. In a recent CDC report, 11.9% of pregnant women between the ages of 18–44 in the US reported current drinking [1]. More recently, a global systematic review and meta-analysis revealed that 9.8% of women worldwide report prenatal alcohol consumption and estimated that 15 children of every 10,000 live births will have fetal alcohol syndrome (FAS), the most severe form of fetal alcohol spectrum disorders FASD [2]. As children with FAS only represent a fraction of children with FASD, FASD are likely the most common preventable cause of neurodevelopmental disabilities worldwide. FASD prevalence estimates range from 2.0–5.0% of the school-age population in the US and Western Europe to 13.6–20.9% in South Africa [3,4]. FASD are characterized by pre- and postnatal growth restriction and a range of neurodevelopmental deficits, and FAS is characterized by a distinctive pattern of facial dysmorphology, small head circumference, and growth restriction [5,6]. Prenatal alcohol exposure (PAE) has been associated with birth defects in all organ systems [6,7,8,9,10,11,12].

There is increasing interest in the potential impact of nutrition on FASD, given concerns regarding potential confounding by poor nutrition among heavy drinkers and the growing body of literature demonstrating the important roles of nutrition as an effect modifier in the setting of maternal alcohol consumption [13,14]. Multiple studies have shown that the diets of heavy-drinking pregnant women in Cape Town, South Africa are similar to the diets of abstainers in their community, indicating that the detrimental effects of PAE found in the region are not confounded by poor maternal nutrition [15,16,17]. These studies did, however, document poor diets among both drinking women and controls across both micro- and macronutrient measures. In a prenatally recruited, prospective longitudinal birth cohort of 206 women, we found that over half of participants reported low food security, and 85% of women reported inadequate intakes of 10 of the 22 nutrients assessed, with minimal differences between heavy drinking women and abstainers/light drinkers [15]. Poor maternal nutrition may have an impact on the teratogenic effects of alcohol given the growing body of literature demonstrating effect modification by nutrition in the setting of PAE. In our prospective longitudinal cohort, we found that fetal alcohol growth restriction was more severe among mothers with poorer nutrition as measured by gestational weight gain and dietary intakes of energy, choline, and iron [13]. In both human and animal studies, choline supplementation has been shown to ameliorate some of the teratogenic effects of alcohol [14,18,19]. In our randomized controlled trial in Cape Town, South Africa, we found that infants born to heavy-drinking mothers receiving high-dose choline supplementation (2 g/d) were more likely to meet eyeblink conditioning criteria, had higher novelty preference scores, indicating better visual recognition memory, and exhibited significant catch-up growth at 6.5 and 12 months than those whose mothers receiving placebo [14]. In animal models, choline deficiency has been shown to exacerbate the teratogenic effects of alcohol [20]. Smith and colleagues have demonstrated that poor maternal iron nutrition may exacerbate fetal alcohol effects on growth and behavior that are ameliorated by maternal iron supplementation [21,22,23].

Little is known regarding the potential impact of postnatal nutrition on FASD. The first two years of life comprise a critical developmental period when micronutrient deficiencies can lead to long-term consequences, such as delayed motor and mental development [24]. In our longitudinal birth cohorts in Detroit [8,25] and Cape Town [7], we found that PAE-related growth restriction worsens during the first year of life but then recovers to the degree seen at birth. Moreover, we also demonstrated that a child’s long-term growth trajectory predicted the severity of PAE-related neurocognitive deficits; those with both fetal and postnatal growth restriction exhibited the most severe deficits, those with only fetal growth restriction, milder deficits, and those with no growth restriction, the mildest [26]. However, to our knowledge, the prospective examination of infant feeding practices in FASD remains unknown.

International infant feeding guidelines recommend that infants should exclusively receive breastmilk for the first 6 months postpartum, followed by the introduction of a diverse diet rich in nutrients to ensure healthy development [24]. Exclusive breastfeeding through 6 months provides all essential nutrients for the baby’s growth (except vitamin D) and has been associated with improved immune function, decreased risk of obesity, and higher IQ [24]. However, little is known about the potential detrimental effects of heavy alcohol use during lactation on the growth and well-being of infants. Some have speculated that the taste of alcohol in breastmilk may lead to decreased infant milk intake. One prospective study found that although infants sucked more frequently when their mothers consumed alcohol, they consumed significantly less milk [27], and other studies have shown that women who drink alcohol during lactation may produce and let-down approximately 20% less milk than mothers who do not drink [28]. Furthermore, little is known about the potential long-term detrimental effects of postnatal alcohol exposure via breastmilk among infants of heavy-drinking mothers. In one retrospective case-control study, children born to women who abstained from alcohol during pregnancy but who drank postnatally while breastfeeding had lower weight percentiles and lower Verbal IQ scores when compared to children whose mothers abstained both pre- and postnatally [29]. Maternal dietary intake of lipids and micronutrients has been shown to alter the composition of breastmilk in the absence of PAE [30,31,32]. Thus, the poor diets seen among heavy-drinking pregnant women in Cape Town may affect postnatal infant nutrition among those who breastfeed.

Given the lack of information regarding the impact of infant nutrition on FASD, we aimed to examine whether infant feeding practices are related to alcohol consumption during pregnancy and may, therefore, potentially play confounding roles in fetal alcohol-related postnatal growth restriction. We also sought to identify the potential effects of postnatal alcohol exposure via breastmilk on growth.

## 2. Materials and Methods

### 2.1. Sample

As we have previously reported, 87 heavy-drinking Cape Colored women and 71 controls were recruited at their first antenatal clinic visit at two midwife obstetric units in peri-urban Cape Town, South Africa between 2011–2015 [33,34]. Alcohol consumption at conception and recruitment was ascertained in timeline follow-back interviews [33,35]. Any woman averaging at least 1.0 oz (30 mL) absolute alcohol (AA)/day or reporting binge drinking (≥2.0 oz (60 mL) AA/drinking occasion) was invited to participate (1 oz (60 mL) AA~ = 1.67 standard drinks). Women who abstained or drank only minimally (with no binge episodes) were invited as controls. Maternal exclusion criteria were: age < 18 years; HIV infection; pharmacological treatment for chronic medical conditions at enrollment. Infant exclusion criteria were: major chromosomal abnormalities; seizures; neural tube defects; very low birthweight (<1500 g); and extreme prematurity (<32 weeks gestation). Consent and interviews were conducted in the mother’s preferred language (Afrikaans or English). Approval was obtained from the Ethics committees at Wayne State University, the University of Cape Town Faculty of Health Sciences, and Columbia University Medical Center.

### 2.2. Ascertainment of Maternal Alcohol, Cigarette Smoking, and Drug Use

In timeline follow-back interviews at recruitment and 4 and 12 weeks thereafter, women were asked about their alcohol use on a day-by-day basis during the previous 2 weeks, with recall linked to specific daily activities [33,35]; women were also interviewed about their cigarette smoking and drug use (cocaine, methamphetamine, opiates, methaqualone, and marijuana; validated by urine ELISA drug testing [9]). Summary prenatal alcohol measures were constructed by averaging across pregnancy: oz AA/day, oz AA/drinking occasion, and frequency of drinking (days/week) [36].

### 2.3. Infant Assessments

Infants were seen at 2 weeks, 6.5-, and 12-month post-partum, corrected for prematurity, and again at age 5 years. The USDA Infant Feeding Questionnaire [37] was modified for application in the present study to assess the following feeding indicators for the first 6.5 months postpartum: breastfeeding practices (e.g., initiation, problems experienced with breastfeeding, duration, reasons for discontinuation if applicable); formula-feeding practices (e.g., type of formula, amount infants drink on average); products added to bottles (vitamins, baby cereal, sweetener, medicine, and iron); age at which formula and/or other foods were first given; and whether the infant received any herbal or botanical preparations since birth. Daily/weekly frequency consumption of the following drinks/solids for the seven days preceding the study was also determined: breastmilk, formula, water, sugar water, cow milk, other milk, other dairy foods, fresh fruit juice, store-bought fruit juice, baby cereal, porridge, desserts (candy, cookies, cake, etc.), biscuits, French fries, peanut butter, bread, pasta, fruit, vegetables, eggs, red meat, chicken, fish/shellfish. Examples of infant formulas and other foods given were adapted to reflect the products used in the target population.

At all four postnatal visits, trained examiners collected infant weight, length, and head circumference measurements using standard WHO procedures, and WHO age- and sex-specific *z*-scores were calculated [38]. As no WHO head circumference normative/percentile data are available at age 5 years, raw cm was used for this outcome.

### 2.4. Postnatal Alcohol Exposure

In timeline follow-back interviews at 6.5 months postpartum, women were asked about their alcohol use [33,35]. Summary postnatal alcohol measures were constructed by averaging across pregnancy: oz AA/day, oz AA/drinking occasion, and frequency of drinking (days/week). As a measure of postnatal alcohol exposure via breastmilk through age 6.5 months, total postnatal AA exposure was calculated as postnatal AA/day × 7 days × number of weeks the infant was breastfed through age 6.5 months.

### 2.5. Demographic and Control Variables

Women were interviewed regarding demographic background, including age, gravidity, and education. Weeks gestation at delivery was determined by early pregnancy ultrasound, if available, or last menstrual period.

### 2.6. Statistical Analyses

Statistical analyses were two-sided (α = 0.05) using SPSS (v.24; IBM, Armonk, NY, USA). All variables were examined for normality of distribution; pre- and postnatal AA/day and total postnatal AA were log-transformed due to skewness (>3.0). Group comparisons between heavy drinking mother-infant pairs and controls were conducted using independent samples *t*-tests for continuous outcomes and chi-square for categorical outcomes. Pearson correlations were used to examine bivariate relations of control variables to specific feeding practices (i.e., weeks breastfeeding, weeks exclusive breastfeeding, weeks formula feeding, weeks complementary feeding) and anthropometry outcomes (Table S1). Linear regression models were constructed to examine the associations of PAE and maternal postnatal alcohol consumption to infant feeding practices and anthropometry measures, and the relations of infant feeding practices to anthropometry measures; multivariable regression models were then constructed to examine these relations controlling for potential confounders. For infant feeding practices, control variables were those related to a given outcome at *p* < 0.10 (Table S1). Control variables for anthropometry outcomes were based on previous studies of fetal alcohol growth restriction [7,8]: maternal age, prenatal cigarettes/day, maternal education, and weeks gestation at delivery. Multivariable linear regression models including potential confounders and both PAE and postnatal alcohol exposure via breastmilk were constructed to compare the relations pre- versus postnatal alcohol exposure to anthropometry measures.

## 3. Results

### 3.1. Sample Characteristics

Sample characteristics and demographics are summarized for heavy-drinking women and controls in Table 1. Women in the cohort were between 18 and 43 years of age and, on average, in their twenties; heavy-drinking women were on average 2 years older than abstainers/light drinkers. Heavy-drinking women were less educated than abstainers/light drinkers, having completed, on average, 0.6 years fewer years of school. Heavy drinking women averaged 7.2 standard drinks/drinking occasion on 1.4 days/week across pregnancy. Of note, heavy-drinking pregnant women reduced their alcohol consumption postnatally, averaging 4.0 drinks/occasion on 1 day/week among the 65.5% who reported postnatal drinking. All but eight controls completely abstained from alcohol during pregnancy, with the eight reporting light drinking with no binges (four women reported one–three drinking occasions across pregnancy; two women reported monthly drinking occasions; one reported drinking 1/week; and one drank 2.1 oz (63 mL) absolute alcohol (~3.5 drinks) on one occasion). Postnatally, 11.3% of controls reported alcohol consumption, averaging 1.2 drinks/occasion on 0.2 days/week. Heavy drinkers were more likely to smoke cigarettes than controls, but the number of cigarettes/days was similar between both groups and generally light. Heavy-drinking pregnant women were also more likely to report marijuana use and used marijuana on an average of 5.7 more days/month. Methamphetamine use was more common among controls than heavy drinking pregnant women.

### 3.2. Infant Feeding Practices

Infant feeding practices are compared between mothers who drank heavily during pregnancy and controls in Table 2 and were similar across the number of total weeks of breastfeeding; weeks exclusive breastfeeding; total weeks formula feeding; weeks exclusive formula feeding; and weeks mixed feeding (both breastmilk and formula). Women who drank heavily during pregnancy reported providing complementary foods for 2.2 fewer weeks than control women. Controls were more than twice as likely to report ‘not enough breastmilk’ as a difficulty with breastfeeding than women who drank heavily during pregnancy. Other difficulties with breastfeeding (e.g., “trouble with latch,” “baby not interested,” and “sore/cracked nipples”) were rare for both groups. Overall, the use of bottle additives was very rare in both groups. One control mother reported adding vitamins to the infant’s bottle vs. none among the heavy exposure group. Among women who drank heavily during pregnancy, two reported adding cereal to their infants’ bottles versus one control mother. No mother in either group reported adding sweetener or iron to the infant’s bottle.

Over 80% of women in both groups reported giving their infants water; sugar water and cow milk were very rarely given, with no group differences (Table 2). Reporting of juice, fruits, vegetables, biscuits, and desserts provision was similar between groups. Women in the heavy exposure group were more likely to report giving their infants porridge (almost 3/4) than controls (just over half). Over half of the heavy exposure group reported feeding their infants eggs vs. less than one-third of control women. Five women (6.9%) in the heavy exposure group reported feeding their infants red meat, whereas no control women reported giving red meat. Over one-third of the mothers in the heavy exposure group reported giving their infant chicken vs. 13.1% of abstainers/light drinkers. Approximately half of the women in the heavy exposure group fed their infants potato fries compared with roughly a quarter of control women.

### 3.3. Relations between Prenatal and Postnatal Maternal Alcohol Consumption and Infant Feeding Practices

Results from regression models demonstrating potential relations of pre- and postnatal alcohol exposure to infant feeding practices are presented in Table 3. Negative trends (*p* < 0.10) were seen between prenatal AA/drinking occasions and weeks exclusive breastfeeding and reporting insufficient breastmilk, while a positive trend was seen with weeks of mixed feeding. Prenatal AA/drinking occasion and drinking frequency were both positively associated with providing chicken and potato fries.

In univariate models, postnatal AA/drinking occasion was negatively associated with weeks complementary foods given, but this relation was no longer seen after adjusting for potential confounders. All three measures of postnatal alcohol consumption were positively associated with giving the infant porridge. Postnatal AA/day and drinking frequency were positively associated with the provision of eggs. Postnatal AA/day was associated with an increased prevalence of feeding the infant chicken. Postnatal average AA/day and drinking frequency were positively associated with the provision of potato fries.

### 3.4. Relations of Prenatal Alcohol Exposure and Infant Feeding Practices to Anthropometry Measures

In Table 4, results from regression models demonstrating the potential relations of prenatal alcohol exposure and infant feeding practices to infant anthropometry outcomes are presented. As expected, all PAE variables were negatively associated with all of the anthropometry measures at 6.5 and 12 months and 5 years. Each week of additional breastfeeding was associated with a 0.1 cm increase in 5-year head circumference after adjusting for potential confounders. Weeks formula feeding was negatively associated with 6.5-month weight *z*-scores in univariate models, but only a trend (*p* < 0.10) was seen in multivariable models. Weeks complementary feeding was positively associated with 5-year weight *z*-scores in univariate but not multivariable models. For any infant feeding practice variable related to an anthropometry measure at *p* < 0.10, we examined whether the relation of PAE to that outcome was independent of the given infant feeding practice variable, and in all cases, the magnitude of the relation of PAE to anthropometry measures was unchanged, and statistical significance was preserved.

### 3.5. Relation of Prenatal Alcohol Exposure and Postnatal Alcohol Exposure to Infant Anthropometry Outcomes

Results from regression models examining potential relations between prenatal alcohol exposure and postnatal alcohol exposure to infant anthropometry outcomes are presented in Table 5. In both univariate models and multivariable models adjusted for potential confounders, total postnatal alcohol exposure through 6.5 months (as measured by postnatal AA/day × 7 days × the number of weeks the infant was breastfed through age 6.5 months) was negatively associated with all anthropometry measures at ages 6.5 and 12 months. When examining multivariable models adjusted for potential confounders for 5-year anthropometry outcomes, only a negative trend (*p* < 0.10) was seen between postnatal alcohol exposure and weight. To examine whether postnatal alcohol exposure was related to growth independent of PAE, we constructed multivariable models including postnatal alcohol exposure, PAE, and potential confounders for each anthropometry outcome. Postnatal alcohol exposure was not related to any anthropometry outcome with control for PAE. Conversely, the relations of PAE to all anthropometry outcomes were virtually unchanged after control for postnatal alcohol exposure.

## 4. Discussion

In this prospective longitudinal cohort of mother-infant pairs in Cape Town, South Africa, infant feeding practices from birth to 6.5 months were very similar between women who drank heavily during pregnancy and abstaining/light-drinking controls. Infant feeding practices were largely unrelated to anthropometry measures at 6.5 and 12 months and 5 years. By contrast and as expected, PAE was related to growth restriction at all ages; these associations were independent of infant feeding practices. Initially, postnatal alcohol exposure appeared to be related to growth restriction during the first year of life, but these relations were no longer observed after control for PAE.

To our knowledge, this is the first study of postpartum infant feeding practices in heavy-drinking pregnant women. As described above, previous studies have documented associations between postnatal alcohol consumption and decreased milk let-down. In a cohort of Taiwanese women, mothers who drank a rice-wine-based soup—each serving the equivalent of 1.071 standard drinks (~14.9 g of alcohol)—reported a longer milk ejection time and less milk volume than those who did not [28]. In contrast, in our cohort of South African mothers, more control women than women from the heavy prenatal drinking group reported not having enough breastmilk as a difficulty with breastfeeding (27.9% vs. 12.5%, respectively), and postnatal alcohol consumption was not related to interview questions regarding difficulty breastfeeding. Heavy-drinking pregnant women reported just 2.2 fewer weeks of complementary feeding and were more likely to give their children food such as porridge, red meat, chicken, eggs, and potato fries, despite having poorer food security, which we have previously reported [40]. For a postpartum feeding practice variable to play confounding roles in the association between PAE and FASD outcomes, the variable must be associated with both exposure and outcome [41]. Our findings of a lack of clinically significant associations between PAE and infant feeding practices thus indicate that the teratogenic effects of alcohol we and others have demonstrated in this community are specific to alcohol and not attributable to poor postnatal nutrition among infants born to mothers who drank heavily during pregnancy.

Accordingly, in the current study, PAE-related growth restriction was independent of infant feeding practices. In longitudinal birth cohorts in both Detroit [8,25] and Cape Town [7], we found that fetal alcohol growth restriction worsens in the first year of life but then recovers to a degree similar to that seen at birth after a period of catch-up growth before age 5 years. As data in that study regarding postnatal nutrition were not available, it was unclear whether this pattern was due to differences in postnatal nutrition or oromotor function among exposed infants or due to the underlying biology of fetal alcohol growth restriction. Consistent with the pattern seen in prior studies, infants born to heavy-drinking pregnant women in this study had, on average, 0.4 smaller length-for-age *z*-scores than control infants at age 2 weeks, followed by 0.8 smaller length-for-age *z*-scores at 6.5 and 12 months; this group difference narrowed to 0.5 *z*-scores at age 5 years—close to the group difference seen at age 2 weeks. The fact that PAE-related growth restriction in this study was independent of infant feeding practices during the first 6.5 months of life suggests that the pattern of worsening growth restriction during infancy is more likely to be due to biological factors than postnatal nutrition. For example, if this pattern were due to oromotor dysfunction, one would expect PAE to have been related to difficulty breastfeeding, which was not seen. Furthermore, the only associations between alcohol and complementary feeding indicated that mothers who drank heavily were more likely to give their children food such as porridge, red meat, chicken, eggs, and potato fries. It should be noted that one limitation of this study is that detailed quantitative data regarding the quantity of breastmilk and/or infant formula and frequency and portion size of each food were not available, so the impact of early postnatal nutrition cannot be fully ruled out. Since data regarding feeding practices beyond 6.5 months were not available, we could not assess the degree to which postnatal nutrition contributed to the postnatal catchup growth seen later in childhood. Of note, chicken and eggs are excellent sources of dietary choline, while red meat and fortified porridge are excellent sources of dietary iron. Given the growing evidence in animal and human studies that dietary choline and iron may mitigate the teratogenic effects of PAE [13,14,20,21,22], it is possible that the provision of these foods among infants with PAE may have blunted some of the effects of PAE and/or postnatal alcohol exposure on growth, and true effect sizes may be even larger in the absence of these foods. Studies examining the impact of postnatal choline and iron in humans are needed to further explore this possibility. Consistent with prior studies demonstrating the beneficial effects of breastfeeding on brain development [24], in this study, each additional week of breastfeeding through 6.5 months was associated with an increase in 5-year head circumference of 0.10 cm. Further studies are needed to assess the potential impact of this breastfeeding-associated increase in head circumference on child neurobehavior.

In multivariable models simultaneously examining associations of both pre- and postnatal alcohol exposure (via breastmilk), postnatal alcohol exposure was not related to any anthropometry measure. Of note, as alcohol is metabolized by the mother’s liver before being excreted in breastmilk, the concentration of alcohol in breastmilk is estimated to be close to the blood alcohol concentration in the mother at the time of breastfeeding [42]. Thus, even though women in this cohort engaged in binge drinking at least weekly, on average, overall ingested postnatal alcohol was likely to be relatively small. Given the weekend-binge pattern of drinking, we could not assess the potential effects of low-level, daily exposure. It must be noted that, as is seen with PAE, postnatal alcohol exposure may affect brain development in the absence of growth restriction. We and others have shown that larger levels of prenatal alcohol exposure are needed to affect growth than are needed to cause brain damage [26,43]; this teratological paradigm could very well hold true in early infancy when brain growth is rapid. Our findings are in contrast to May et al.’s study, in which children with postnatal alcohol exposure in the absence of PAE had lower 7-year weight percentiles than children with neither pre- nor postnatal alcohol exposure [29]. A potentially real but small effect of postnatal alcohol exposure on growth may have been difficult to detect in our cohort, in which PAE levels were quite high, and few children had strictly pre- or postnatal alcohol exposure. It is possible that mothers in the May et al. study drank more frequently and/or in higher quantities than mothers in our cohort. It is also possible that postnatal alcohol exposure for a longer period of time is needed to demonstrate effects on growth, and our breastfeeding data were limited to 6.5 months. However, since for the vast majority of infants, the quantity of breastmilk consumed per day rapidly decreases after age 6 months, as complementary feeding replaces breastmilk as the major source of nutrition, the degree of continued postnatal alcohol exposure after age 6.5 months was likely to be quite small. As the May et al. [29] study was retrospective, and postnatal alcohol exposure was also associated with smaller palpebral fissures and higher dysmorphology score outcomes that are likely biologically set in utero, some degree of apparent postnatal alcohol-associated growth restriction may have been attributable to prenatal exposure that was not fully adjusted for due to recall errors in retrospective interviews.

## 5. Conclusions

In this prospective, longitudinal birth cohort, infant feeding practices in the first 6.5 months postpartum between mothers who drank heavily during pregnancy were very similar to those among women who abstained or drank at very light levels, indicating that the teratogenic effects of alcohol we and others have documented in this population are unlikely to be confounded by postnatal feeding practices in the first 6.5 months of life. Consistent with this principle, fetal alcohol growth restriction was independent of infant feeding practices; future studies are needed to determine whether PAE-related neurobehavioral deficits are also independent of postnatal feeding. We confirmed our previous findings of fetal alcohol growth restriction seen at birth, worsening over the first year of life, and returning to the degree seen at birth by age 5 years. We also found that, with control for PAE, postnatal alcohol exposure was not associated with growth restriction. Future studies are needed to examine the potential impact of postnatal alcohol exposure on neurodevelopment to inform breastfeeding recommendations for women who consume alcohol in the postnatal period.

## Figures and Tables

**Table 1:** Sample characteristics.

	Controls (*n* = 71)		Heavy Drinking Mothers (*n* = 87)		
	*n*	*M* (SD) or *n* (%)	*n*	*M* (SD) or *n* (%)	*p* ^a^
Maternal characteristics					
Maternal age at conception (years)	71	25.5 (4.9)	87	27.9 (6.1)	0.003
Gravidity (no. previous pregnancies (%))	71	1.5 (1.3)	87	1.8 (1.5)	0.090
Education (yr. school completed)	70	9.9 (1.7)	87	9.3 (1.6)	0.007
Marital status, no. married (%)	71	30 (42.3)	87	25 (28.7)	0.076
Prenatal alcohol and drug use					
Prenatal alcohol consumption:	71		87		
oz AA/day		0.0 (0.0)		0.9 (1.2)	<0.001
oz AA/drinking day		0.1 (0.4)		4.3 (2.4)	<0.001
Drinking days/week		0.0 (0.1)		1.4 (1.1)	<0.001
No. (%) reporting cigarette smoking	71	49 (69.0)	87	71 (81.6)	0.065
Cigarettes/day among smokers	49	5.8 (3.8)	71	6.7 (4.4)	0.121
No. (%) reporting marijuana use	71	8 (11.3)	87	18 (20.7)	0.026
Marijuana days/month among users	8	4.0 (4.7)	18	9.7 (9.0)	0.047
No. (%) reporting methamphetamine use	71	11 (15.5)	87	12 (13.8)	0.763
Methamphetamine days/month among users	11	10.2 (8.9)	12	4.3 (5.4)	0.033
Postnatal alcohol consumption (at 6.5 months):					
No. (%) reporting drinking	71	8 (11.3)	84	55 (65.5)	<0.001
oz AA/day	8	0.0 (0.0)	55	0.3 (0.3)	<0.001
oz AA/drinking day	8	0.7 (0.3)	55	2.4 (1.0)	<0.001
Drinking days/week	8	0.2 (0.3)	55	1.0 (0.8)	<0.001
Infant characteristics					
Weeks gestation at delivery	71	39.1 (1.8)	87	38.8 (2.1)	0.223
Preterm delivery (<37 weeks), no. (%)	71	9 (12.7)	87	12 (13.8)	0.837
Infant sex, no. female (%)	71	33 (46.5)	87	45 (51.7)	0.512
Infant birthweight (g)	70	3078.8 (534.5)	87	2885.4 (609.5)	0.019
No. small for gestational age (%) ^b^	70	13 (18.6)	84	30 (35.7)	0.018
2-week *z*-scores ^c^					
Length	61	−1.3 (1.3)	69	−1.7 (1.4)	0.083
Weight	61	−0.8 (1.2)	69	−1.3 (1.4)	0.029
Head circumference	61	−0.4 (1.2)	69	−0.9 (1.5)	0.016
6.5-month *z*-scores ^c^					
Length	71	−0.7 (1.0)	83	−1.58 (1.1)	<0.001
Weight	71	0.1 (0.9)	83	−0.9 (1.2)	<0.001
Head circumference	71	−0.1 (0.9)	83	−0.9 (1.0)	<0.001
12-month *z*-scores ^c^					
Length	68	−0.8 (0.9)	81	−1.6 (1.1)	<0.001
Weight	69	0.1 (0.9)	80	−0.9 (1.0)	<0.001
Head circumference	69	−0.2 (0.9)	81	−1.0 (0.9)	<0.001
5-year *z*-scores ^c^					
Height	67	−0.5 (0.9)	80	−1.0 (0.9)	0.005
Weight	67	−0.2 (1.0)	80	−0.9 (0.8)	<0.001
5-year head circumference (cm)	67	50.3 (1.6)	80	48.7 (3.4)	<0.001

AA = absolute alcohol; 1 oz AA = 30 mL AA = 1.67 standard drinks. AA = absolute alcohol; 1 oz AA = 30 mL AA = 1.67 standard drinks. ^a^ From independent samples *t*-tests for continuous outcomes, chi-square for binary outcomes. ^b^ Defined as birthweight < 10th percentile for gestational age [39]. ^c^ Age- and sex-specific *z*-scores from World Health Organization norms [38].

**Table 2:** Infant feeding practices, breastfeeding difficulty, bottle additives, and complementary foods.

		Controls (*n* = 61)				Heavy Drinking Women (*n* = 72)					
	*n*	M	SD	*n*	%	*n*	M	SD	*n*	%	*p* ^a^
Weeks breastfed	61	26.9	7.6			72	26.4	7.3			0.354
Weeks formula fed	61	8.5	10.8			72	10.5	10.7			0.151
Weeks complementary food given	61	3.9	7.8			72	1.7	5.5			0.035
Weeks exclusively breastfed	61	19.5	10.1			72	17.2	10.4			0.103
Weeks exclusively formula fed	61	2.1	7.3			72	2.2	6.9			0.486
Weeks mixed fed	61	7.5	9.3			72	9.3	9.9			0.144
Difficulty breastfeeding	61					72					
Trouble with latch, no. (%)				1	1.6				0	0.0	0.279
Baby not interested, no. (%)				0	0.0				1	1.4	0.359
Not enough breastmilk, no. (%)				17	27.9				9	12.5	0.026
Sore/cracked nipples, no. (%)				1	2.2				0	0.0	0.235
Bottle Additives											
Vitamins, no. (%) adding	61			1	1.6	72			0.0	0.0	0.275
Cereal, no. (%) adding	61			1	1.6	72			2.0	2.0	0.275
Sweetener, no. (%) adding	61			0	0.0	72			0	0.0	--
Medicine, no. (%) adding	61			0	0.0	72			0	0.0	--
Iron, no. (%) adding	61			0	0.0	72			0	0.0	--
Complementary feeding											
Water, no. (%) given	61			50	82.0	72			63	87.5	0.525
Water days/week (users only)		1.2	0.7				1.1	0.4			0.333
Sugar water, no. (%) given	61			1	1.6	72			0	0.0	0.275
Sugar water days/week (users only)		1	--				--	--			--
Cow’s milk, no. (%) given	61			1	1.6	72			--	--	0.208
Cow’s milk days/week (users only)		2.0	--				--	--			--
Juice, no. (%) given	61			9	14.8	72			14	19.4	0.476
Juice days/week (users only)		0.6	0.4				0.9	0.6			0.257
Porridge, no. (%) given	61			32	52.5	72			52	72.2	0.019
Porridge days/week (users only)		1.0	0.2				2.5	10.0			0.194
Biscuits, Bread or Pasta, no. (%) given	61			4	6.6	72			8	11.1	0.361
Biscuits, etc. days/week (users only)		1.3	1.2				1.0	0.0			0.623
Fruits, no. (%) given	61			23	37.7	72			33	45.8	0.344
Fruits days/week (users only)		0.9	0.5				1.1	0.6			0.086
Vegetables, no. (%) given	61			52	85.2	72			66	91.7	0.243
Vegetables days/week (users only)		1.3	1.6				1.4	1.9			0.385
Desserts, no. (%) given	61			45	73.8	72			49	68.1	0.471
Desserts days/week (users only)		0.5	0.3				0.5	0.3			0.536
Eggs, no. (%) given	61			18	29.5	72			41	56.9	0.002
Eggs days/week (users only)		0.2	0.1				0.3	0.2			0.191
Red meat, no. (%) given	61			0	(0.0)	72			5	6.9	0.036
Red meat days/week (users only)		--	--				0.8	0.4			--
Chicken, no. (%) given	61			8	13.1	72			25	34.7	0.004
Chicken days/week (users only)		0.3	0.1				0.3	0.2			0.491
Fish, no. (%) given	61			3	4.9	72			4	5.6	0.870
Fish days/week (users only)		0.1	0.0				0.3	0.1			0.093
Fries, no. (%) given	61			14	23.0	72			40	55.6	<0.001
Fries days/week (users only)		0.3	0.1				0.2	0.1			0.453

^a^ From independent samples *t*-tests for continuous outcomes, chi-square for binary outcomes.

**Table 3:** Relation of pre- and postnatal alcohol exposure to infant feeding practices.

	Prenatal Alcohol Exposure						Postnatal Alcohol Exposure through 6.5 mo.					
	AA/Day (Logged Values)		AA/Drinking Occasion		Drinking Frequency		AA/Day		AA/ Drinking Occasion		Drinking Frequency	
	*Β*_1_	*Β*_2_	*Β*_1_	*Β*_2_	*Β*_1_	*Β*_2_	*Β*_1_	*Β*_2_	*Β*_1_	*Β*_2_	*Β*_1_	*Β*_2_
Infant feeding practices												
Weeks breastfed	0.17 (−3.17, 3.50)	0.22 (−3.08, 3.53)	−0.02 (−0.47, 0.44)	−0.03 (−0.43, 0.48)	0.12 (−1.29, 1.53)	0.11 (−1.29, 1.51)	3.26 (−4.58, 11.10)	3.31 (−4.46, 11.1)	0.20 (−0.83, 1.23)	0.12 (−0.91, 1.15)	0.82 (−1.37, 3.01)	0.82 (−1.35, 2.98)
Weeks formula fed	2.95 (−1.84, 7.74)	2.97 (−1.76, 7.70)	0.47 (−0.18, 1.11)	0.47 (−0.17, 1.11)	0.79 (−1.24, 2.82)	0.75 (−1.26, 2.76)	2.31 (−9.03, 13.64)	1.90 (−9.32, 13.11)	0.68 (−0.81, 2.16)	0.60 (−0.88, 2.08)	−0.52 (−2.65, 3.68)	0.31 (−2.84, 3.46)
Weeks complementary foods	−1.50 (−4.49, 1.49)	−1.34 (−4.30, 1.62)	−0.19 (−0.60, 0.21)	−0.13 (−0.54, 0.27)	−0.87 (−2.13, 0.39)	−0.82 (−2.07, 0.42)	−5.01 (−12.02, 2.00)	−4.25 (−11.24, 2.74)	−0.92 * (−1.84, −0.01)	−0.76 (−1.69, 0.17)	−1.47 (−3.42, 0.49)	−1.27 (−3.22, 0.68)
Weeks exclusively breastfed	−3.22 (−7.80, 1.36)	−3.20 (−7.74, 1.34)	−0.55 ^†^ (−1.17, 0.07)	−0.54 ^†^ (−1.16, 0.07)	−0.76 (−2.70, 1.19)	−0.70 (−2.64, 1.23)	−1.42 (−12.29, 9.45)	−0.94 (−11.74, 9.85)	−0.54 (−1.96, 0.89)	−0.45 (−1.87, 0.98)	−0.14 (−3.17, 2.90)	0.09 (−2.93, 3.12)
Weeks exclusively formula fed	−0.38 (−3.53, 2.76)	--	−0.04 (−0.46, 0.39)	--	−0.17 (−1.49, 1.16)	--	−2.33 (−9.72, 5.06)	--	−0.15 (−1.12, 0.82)	--	−0.54 (−2.61, 1.52)	--
Weeks mixed feeding	3.39 (−0.88, 7.65)	--	0.53 ^†^ (−0.05, 1.11)	--	0.88 (−0.93, 2.69)	--	4.68 (−5.43, 14.78)	--	0.74 (−0.59, 2.06)	--	0.96 (−1.87, 3.78)	--
Reporting insufficient breastmilk	−0.11 (−0.29, 0.07)	--	−0.02 ^†^ (−0.05, 0.00)	--	−0.04 (−0.11, 0.04)	--	−0.12 (−0.54, 0.30)	--	−0.03 (−0.08, 0.03)	--	−0.01 (−0.13, 0.11)	--
Complementary food provision		--		--		--		--		--		--
Juice	0.10 (−0.07, 0.27)	--	0.00 (−0.02, 0.03)	--	0.06 (−0.01, 0.13)	--	0.14 (−0.26, 0.54)	--	0.00 (−0.06, 0.05)	--	0.07 (−0.04, 0.19)	--
Porridge	0.15 (−0.07, 0.36)	--	0.02 ^†^ (−0.01, 0.05)	--	0.07 (−0.02, 0.16)	--	0.67 ** (0.17, 1.17)	--	0.09 ** (0.02, 0.15)	--	0.19 ** (0.05, 0.33)	--
Fruits	0.07 (−0.15, 0.30)	--	0.01 (−0.02, 0.04)	--	0.04 (−0.05, 0.14)	--	−0.04 (−0.56, 0.48)	--	0.00 (−0.07, 0.07)	--	0.01 (−0.14, 0.15)	--
Vegetables	0.08 (−0.06, 0.22)	0.09 (−0.05, 0.23)	0.08 (−0.06, 0.22)	0.01 (−0.01, 0.03)	0.01 (−0.01, 0.03)	0.04 (−0.02, 0.10)	0.24 (−0.10, 0.57)	0.28 ^†^ (−0.04, 0.61)	0.03 (−0.02, 0.07)	0.04 ^†^ (0.00, 0.09)	0.06 (−0.03, 0.16)	0.08 ^†^ (−0.02, 0.17)
Eggs	0.11 (−0.11, 0.33)	--	0.03 ^†^ (0.00, 0.06)	--	0.06 (−0.04, 0.15)	--	0.46 ^†^ (−0.06, 0.98)	--	0.08 * (0.01, 0.15)	--	0.15 * (0.00, 0.29)	--
Chicken	0.20 * (0.01, 0.39)	0.15 (−0.05, 0.35)	0.04 ** (0.01, 0.06)	0.03 * (0.00, 0.06)	0.10 * (0.02, 0.18)	0.08 * (0.00, 0.17)	0.31 (−0.14, 0.77)	0.17 (−0.30, 0.64)	0.08 ** (0.02, 0.14)	0.06 * (0.00, 0.13)	0.12 ^†^ (0.00, 0.25)	0.09 (−0.04, 0.22)
Fries	0.24 * (0.02, 0.46)	--	0.04 ** (0.01, 0.07)	--	0.11 * (0.02, 0.20)	--	0.59 * (0.08, 1.10)	--	0.06 ^†^ (−0.01, 0.13)	--	0.16 * (0.01, 0.30)	--

*Β*_1_ = regression coefficient from univariate models regressing the outcome on the given alcohol variable; *Β*_2_ = regression coefficient from multivariable models regressing the outcome on the given alcohol variable and potential confounders related to the outcome at *p* < 0.10 in Pearson correlations (see Table S1, weeks breastfed: weeks gestation at delivery; weeks formula fed: highest school grade completed by mother; weeks complementary foods: prenatal cigarettes/day; weeks exclusive breastfeeding: highest school grade completed by mother; vegetables given (yes/no): prenatal cigarettes/day; chicken given (yes/no): maternal age, highest school grade completed by mother). ^†^ *p* < 0.10; * *p* < 0.05; ** *p* < 0.01.

**Table 4:** Relation of prenatal alcohol exposure and infant feeding practices to infant anthropometry outcomes.

	Prenatal Alcohol Exposure				Infant Feeding Practices										Prenatal Alcohol Exposure with Control for Infant Feeding
	AA/Drinking Occasion	Drinking Frequency	AA/Day (Logged Values)		Weeks Breastfeeding		Weeks Formula Feeding		Weeks Complementary Feeding		Weeks Exclusive Breastfeeding		Weeks Mixed (Breastmilk and Formula) Feeding		AA/Day (Logged Values)
	*Β*_1_	*Β*_1_	*Β*_1_	*Β*_2_	*Β*_1_	*Β*_2_	*Β*_1_	*Β*_2_	*Β*_1_	*Β*_2_	*Β*_1_	*Β*_2_	*Β*_1_	*Β*_2_	*Β*_3_
6.5-month anthropometry *z*-scores ^a^ (*n* = 71 controls, 83 heavily exposed)															
Length	−0.14 *** (−0.20, −0.08)	−0.42 *** (−0.59, −0.25)	−1.08 *** (−1.52, −0.65)	−0.97 *** (−1.35, −0.59)	0.00 (−0.03, 0.03)	0.01 (−0.01, 0.03)	−0.02 * (−0.04, 0.00)	−0.02 ^†^ (−0.03, 0.00)	0.01 (−0.02, 0.04)	0.01 (−0.02, 0.03)	0.01 (−0.01, 0.03)	0.01 (−0.01, 0.03)	−0.01 (−0.03, 0.01)	−0.01 (−0.02, 0.01)	−0.93 ***^b^ (−1.35, −0.52)
Weight	−0.15 *** (−0.21, −0.08)	−0.32 *** (−0.50, −0.14)	−0.94 *** (−1.39, −0.48)	−0.75 *** (−1.17, −0.33)	0.00 (−0.02, 0.03)	0.01 (−0.01, 0.04)	−0.02 ^†^ (−0.03, 0.00)	−0.02 (−0.03, 0.01)	0.01 (−0.02, 0.04)	0.00 (−0.03, 0.03)	0.01 (−0.01, 0.03)	0.01 (−0.01, 0.03)	−0.01 (−0.03, 0.01)	0.00 (−0.02, 0.02)	−0.71 **^b^ (−1.16, −0.25)
Head circumference	−0.15 *** (−0.19, −0.07)	−0.32 *** (−0.48, −0.17)	−0.91 *** (−1.30, −0.52)	−0.78 *** (−1.14, −0.41)	−0.01 (−0.03, 0.01)	0.00 (−0.02, 0.02)	−0.01 (−0.02, 0.01)	0.00 (−0.01, 0.02)	0.01 (−0.02, 0.04)	0.01 (−0.01, 0.03)	0.00 (−0.02, 0.02)	0.00 (−0.02, 0.01)	−0.01 (−0.03, 0.01)	0.00 (−0.02, 0.02)	--
12-month anthropometry *z*-scores ^a^ (*n* = 69 controls, 81 heavily exposed)															
Length	−0.13 *** (−0.18, −0.06)	−0.43 *** (−0.59, −0.28)	−1.06 *** (−1.46, −0.66)	−0.94 *** (−1.30, −0.59)	−0.01 (−0.03, 0.02)	0.01 (−0.02, 0.03)	−0.01 (−0.03, 0.01)	−0.01 (−0.02, 0.01)	0.02 (−0.01, 0.05)	0.01 (−0.01, 0.04)	0.00 (−0.01, 0.02)	0.01 (−0.01, 0.02)	−0.01 (−0.03, 0.01)	0.00 (−0.02, 0.02)	--
Weight	−0.15 *** (−0.21, −0.09)	−0.35 *** (−0.51, −0.18)	−1.00 *** (−1.41, −0.58)	−0.91 *** (−1.32, −0.51)	0.00 (−0.03, 0.02)	0.00 (−0.02, 0.03)	0.00 (−0.02, 0.02)	0.00 (−0.01, 0.02)	0.03 ^†^ (0.00, −0.05)	0.02 (−0.01, 0.05)	−0.01 (−0.02, 0.01)	−0.01 (−0.02, 0.01)	000 (−0.02, 0.02)	0.01 (−0.01, 0.03)	−0.88 ***^c^ (−1.33, −0.43)
Head circumference	−0.14 *** (−0.19, −0.08)	−0.35 ** (−0.50, −0.20)	−0.90 *** (−1.28, −0.52)	−0.83 *** (−1.20, −0.46)	−0.02 (−0.05, 0.01)	−0.01 (−0.03, 0.02)	0.01 (−0.01, 0.02)	0.01 (−0.01, 0.03)	0.02 (−0.01, 0.04)	0.02 (−0.01, 0.04)	−0.01 (−0.03, 0.01)	−0.01 (−0.03, 0.01)	0.00 (−0.02, 0.02)	0.01 (−0.01, 0.03)	--
5-year anthropometry *z*-scores ^a^ (*n* = 67 controls, 80 heavily exposed)															
Height	−0.05 * (−0.11, 0.00)	−0.24 *** (−0.38, −0.10)	−0.54 ** (−0.90, −0.19)	−0.48 *** (−0.84, −0.12)	0.01 (−0.02, 0.03)	0.01 (−0.01, 0.04)	−0.01 (−0.02, 0.01)	0.00 (−0.02, 0.01)	0.02 ^†^ (0.00, 0.04)	0.02 (−0.01, 0.04)	0.00 (−0.02, 0.02)	0.00 (−0.02, 0.01)	0.00 (−0.01, 0.02)	0.01 (−0.01, 0.03)	−0.50 *^c^ (−0.90, −0.09)
Weight	−0.09 *** (−0.15, −0.04)	−0.29 *** (−0.43, −0.15)	−0.71 *** (−1.08, −0.35)	−0.60 *** (−0.95, −0.25)	0.01 (−0.02, 0.03)	0.01 (−0.01, 0.04)	0.00 (−0.02, 0.01)	0.00 (−0.01, 0.01)	0.03 * (0.00, 0.05)	0.02 (−0.01, 0.04)	0.00 (−0.02, 0.02)	0.00 (−0.02, 0.01)	0.01 (−0.01, 0.02)	0.01 (−0.01, 0.03)	−0.62 **^c^ (−1.02, −0.22)
5-year Head circumference (cm)	−0.22 ** (−0.37, −0.06)	−0.70 *** (−1.13, −0.28)	−1.58 ** (−2.67, −0.49)	−1.70 ** (−2.84, −0.57)	0.08 * (0.00, 0.15)	0.10 * (0.03, 0.18)	−0.02 (−0.07, 0.03)	−0.02 (−0.07, 0.03)	0.05 (−0.03, 0.13)	0.06 (−0.02, 0.14)	0.02 (−0.03, 0.07)	0.02 (−0.03, 0.08)	0.02 (−0.04, 0.07)	0.03 (−0.03, 0.08)	−1.48 *^d^ (−2.79, −0.17)

*Β*_1_ = regression coefficient from univariate model regressing the outcome on the given alcohol variable; *Β*_2_ = regression coefficient from multivariable model regressing the outcome on the given alcohol variable and potential confounders chosen based on prior studies (maternal age, prenatal cigarettes/day, maternal education, and weeks gestation at delivery for anthropometry measures, with the addition of age at time of measurement for 5-year head circumference); *Β*_3_ = regression coefficient from multivariable model regressing the outcome on the given alcohol variable, the named infant feeding variable (related to the given outcome at *p* < 0.10 in univariate models) and potential confounders. ^a^ Age- and sex-specific z-scores from World Health Organization norms [38]. ^b^ Adjusted for weeks formula feeding. ^c^ Adjusted for weeks complementary feeding. ^d^ Adjusted for weeks breastfeeding. ^†^ *p* < 0.10; * *p* < 0.05; ** *p* < 0.01; *** *p* < 0.001

**Table 5:** Relation of prenatal alcohol exposure and postnatal alcohol exposure to infant anthropometry outcomes.

	Prenatal Alcohol Exposure (AA/Day, Logged Values)			Postnatal Alcohol Exposure through 6.5 Months (Total AA ^a^, Logged Values)		
	Univariate	Multivariable	Multivariable with Control for Postnatal Total AA ^a^	Univariate	Multivariable	Multivariable with Control for Prenatal AA/Day
	*Β*_1_	*Β*_2_	*Β*_3_	*Β*_1_	*Β*_2_	*Β*_3_
6.5-month anthropometry *z*-scores ^b^ (*n* = 71 controls, 83 heavily exposed)						
Length	−1.08 *** (−1.52, −0.65)	−0.97 *** (−1.35, −0.59)	−1.07 *** (−1.59, −0.55)	−0.28 ** (−0.45, −0.10)	−0.22 ** (−0.38, −0.06)	0.03 (−0.16, 0.23)
Weight	−0.94 *** (−1.39, −0.48)	−0.75 *** (−1.17, −0.33)	−0.87 ** (−1.44, −0.29)	−0.25 ** (−0.43, −0.08)	−0.18 * (−0.35, −0.01)	0.03 (−0.19, 0.24)
Head circumference	−0.91 *** (−1.30, −0.52)	−0.78 *** (−1.14, −0.41)	−0.83 ** (−1.34, −0.32)	−0.22 ** (−0.38, −0.06)	−0.16 * (−0.31, −0.01)	0.04 (−0.15, 0.23)
12-month anthropometry *z*-scores ^b^ (*n* = 69 controls, 81 heavily exposed)						
Length	−1.06 *** (−1.46, −0.66)	−0.94 *** (−1.30, −0.59)	−1.01 *** (−1.49, −0.54)	−0.25 ** (−0.41, −0.09)	−0.19 ** (−0.34, −0.05)	0.05 (−0.13, 0.23)
Weight	−1.00 *** (−1.41, −0.58)	−0.91 *** (−1.32, −0.51)	−0.87 ** (−1.43, −0.32)	−0.29 *** (−0.45, −0.12)	−0.25 ** (−0.42, −0.09)	−0.04 (−0.25, 0.16)
Head circumference	−0.90 *** (−1.28, −0.52)	−0.83 *** (−1.20, −0.46)	−0.81 ** (−1.32, −0.30)	−0.21 ** (−0.37, −0.05)	−0.17 * (−0.33, −0.02)	0.02 (−0.17, 0.21)
5-year anthropometry *z*-scores ^b^ (*n* = 67 controls, 80 heavily exposed)						
Height	−0.54 ** (−0.90, −0.19)	−0.48 *** (−0.84, −0.12)	−0.62 * (−1.14, −0.11)	−0.13 ^†^ (−0.28, 0.02)	−0.10 (−0.25, 0.06)	0.06 (−0.14, 0.25)
Weight	−0.71 *** (−1.08, −0.35)	−0.60 *** (−0.95, −0.25)	−0.74 ** (−1.24, −0.24)	−0.19 * (−0.35, −0.04)	−0.13 ^†^ (−0.29, 0.02)	0.04 (−0.15, 0.24)
5-year Head circumference (cm)	−1.58 ** (−2.67, −0.49)	−1.70 ** (−2.84, −0.57)	−2.12 * (−3.78, −0.47)	−0.17 (−0.63, 0.30)	−0.18 (−0.68, 0.31)	0.33 (−0.30, 0.96)

*Β*_1_ = regression coefficient from univariate model regressing the outcome on the given alcohol variable; *Β*_2_ = regression coefficient from multivariable model regressing the outcome on the given alcohol variable and potential confounders chosen based on prior studies (maternal age, prenatal cigarettes/day, maternal education, and weeks gestation at delivery for anthropometry measures, with the addition of age at time of measurement for 5-year head circumference); *Β*_3_ = regression coefficient from multivariable model regressing the outcome on prenatal AA/day (logged values), postnatal total AA ^a^ (logged values), and potential confounders. ^a^ Total postnatal AA = postnatal AA/day × 7 days × the number of weeks the infant was breastfed through age 6.5 months. ^b^ Age- and sex-specific z-scores from World Health Organization norms [38]. ^†^ *p* < 0.10; * *p* < 0.05; ** *p* < 0.01; *** *p* < 0.001.

## Data Availability

De-identified, individual participant data that underlie the results reported in this article and the study protocol, statistical analysis plan, and analytic code will be available for sharing with journal editors for any reason either before or after publication for checking and to researchers who provide a methodologically sound proposal, as determined by the authors of this article. Proposals from interested parties should be directed to Sandra W. Jacobson, PhD (sandra.jacobson@wayne.edu). Data will be stored in a data repository at Wayne State University and transmitted electronically in encrypted form to requestors. Data requestors will need to sign a data access agreement prior to access.

## Supplementary Materials

The following supporting information can be downloaded at: https://www.mdpi.com/article/10.3390/nu15092018/s1: Table S1. Univariate relations between control variables and infant feeding practices.
