# Supplementary material for: Fetal Alcohol-Related Postnatal Growth Restriction Is Independent of Infant Feeding Practices and Postnatal Alcohol Exposure in a Prospective South African Birth Cohort
Source: Nutrients. 2023 Apr 22;15(9):2018. doi: 10.3390/nu15092018 (PMC10181362; doi:10.3390/nu15092018)
Supplement: Supplementary file 1 [file nutrients-15-02018-s001.zip › nutrients-2306346-supplementary.pdf]

**Supplementary Materials for:**

**Fetal alcohol growth restriction is independent of infant feeding practices and postnatal alcohol exposure in a prospective South African birth cohort**

Alexia C. Edwards, Sandra W. Jacobson, Marjanne Senekal, Neil C. Dodge, Christopher D. Molteno, Ernesta M. Meintjes, Joseph L. Jacobson, R. Colin Carter

**Table S1. Univariate relations between control variables and infant feeding practices**[a]

| | Maternal age | Gravidity | Maternal education | Cigarettes/ day | Days/mo marijuana use | Days/mo metham-phetamine use | Weeks gestation at delivery | Infant sex |
|---|---|---|---|---|---|---|---|---|
| <u>Infant feeding practices</u> | | | | | | | | |
| Weeks breastfed | 0.06 | 0.01 | 0.04 | -0.08 | -0.03 | -0.01 | -0.16† | 0.09 |
| Weeks formula fed | -0.01 | -0.03 | -0.15† | 0.04 | -0.06 | 0.01 | 0.03 | -0.06 |
| Weeks complementary foods | -0.08 | -0.12 | 0.12 | -0.18* | -0.05 | 0.02 | 0.04 | -0.03 |
| Weeks exclusively breastfed | 0.00 | 0.04 | 0.15† | -0.04 | 0.08 | 0.03 | -0.12 | 0.04 |
| Weeks exclusively formula fed | -0.06 | -0.02 | -0.01 | 0.07 | 0.07 | 0.04 | 0.08 | -0.09 |
| Weeks mixed feeding | 0.04 | -0.04 | -0.13 | -0.02 | -0.11 | -0.03 | 0.01 | 0.03 |
| Reporting insufficient breastmilk | -0.09 | -0.08 | -0.11 | 0.07 | -0.05 | 0.14 | -0.08 | -0.03 |
| <u>Complementary food provision</u> | | | | | | | | |
| Juice | -0.09 | -0.08 | 0.03 | 0.031 | -0.02 | -0.07 | 0.06 | 0.01 |
| Porridge | 0.04 | 0.10 | -0.14 | -0.04 | -0.08 | -0.01 | -0.08 | -0.13 |
| Fruits | -0.04 | -0.03 | -0.01 | -0.11 | 0.05 | 0.01 | 0.03 | 0.02 |
| Vegetables | -0.01 | -0.06 | -0.04 | -0.21* | -0.09 | 0.09 | 0.14 | -0.03 |
| Eggs | 0.04 | 0.13 | -0.01 | 0.06 | -0.04 | -0.11 | -0.11 | 0.01 |
| Chicken | 0.17† | 0.14 | -0.15† | -0.05 | -0.08 | -0.04 | -0.07 | -0.11 |
| Fries | -0.05 | -0.09 | -0.07 | -0.02 | 0.07 | -0.01 | 0.00 | 0.11 |

†$p < 0.10$; *$p < 0.05$; **$p < 0.01$; ***$p < 0.001$.

[a]Values are Pearson *r*'s
